# Exogenous H_2_S prevents the nuclear translocation of PDC‐E1 and inhibits vascular smooth muscle cell proliferation in the diabetic state

**DOI:** 10.1111/jcmm.16688

**Published:** 2021-08-21

**Authors:** Linxue Zhang, Xiaoshu Jiang, Ning Liu, Mingyu Li, Jiaxin Kang, Lingxue Chen, Jingyuan Tang, Shiyun Dong, Fanghao Lu, Weihua Zhang

**Affiliations:** ^1^ Department of Pathophysiology Harbin Medical University Harbin China; ^2^ Department of Functional experiment center Harbin Medical University Harbin China

**Keywords:** diabetes mellitus, hydrogen sulphide (H_2_S), pyruvate dehydrogenase complex‐E1 (PDC‐E1), vascular smooth muscle cell

## Abstract

Hydrogen sulphide (H_2_S) inhibits vascular smooth muscle cell (VSMC) proliferation induced by hyperglycaemia and hyperlipidaemia; however, the mechanisms are unclear. Here, we observed lower H_2_S levels and higher expression of the proliferation‐related proteins PCNA and cyclin D1 in db/db mouse aortae and vascular smooth muscle cells treated with 40 mmol/L glucose and 500 μmol/L palmitate, whereas exogenous H_2_S decreased PCNA and cyclin D1 expression. The nuclear translocation of mitochondrial pyruvate dehydrogenase complex‐E1 (PDC‐E1) was significantly increased in VSMCs treated with high glucose and palmitate, and it increased the level of acetyl‐CoA and histone acetylation (H3K9Ac). Exogenous H_2_S inhibited PDC‐E1 translocation from the mitochondria to the nucleus because PDC‐E1 was modified by S‐sulfhydration. In addition, PDC‐E1 was mutated at Cys101. Overexpression of PDC‐E1 mutated at Cys101 increased histone acetylation (H3K9Ac) and VSMC proliferation. Based on these findings, H_2_S regulated PDC‐E1 S‐sulfhydration at Cys101 to prevent its translocation from the mitochondria to the nucleus and to inhibit VSMC proliferation under diabetic conditions.

## 1 INTRODUCTION

Vascular complications, such as atherosclerosis, in individuals with diabetes mellitus (DM) increase patient morbidity and mortality.^1^ As a main component of the artery wall, the proliferation of vascular smooth muscle cells (VSMCs) plays a pivotal role in the initiation and development of diabetic vascular complications.^2^ Recent studies have confirmed that chronic hyperglycaemia/high glucose (HG)–enhanced reactive oxygen species (ROS) production accelerates the progression of VSMC proliferation.^3^ However, the mechanisms of VSMC proliferation have not been completely explained. Due to the complexity of pathogenic mechanisms, new insights are required to explore hyperglycaemia/high glucose (HG)–induced VSMC proliferation. Based on accumulating evidence, epigenetic regulation of gene expression is essential for cell proliferation and differentiation through the modifications of core histones by methylation, phosphorylation and acetylation.^4^ In metazoan cells, the biosynthesis of acetyl‐coenzyme A (CoA) in different subcellular compartments, such as in mitochondria and the nucleus, has been affirmed. ATP citrate lyase (ACL) has been reported to be present in the nucleus. ACL, the main enzyme for nuclear acetyl‐CoA generation in mammalian cells, utilizes mitochondrial citrate as its substrate.^5^ According to recent studies, the pyruvate dehydrogenase complex (PDC) might be translocated from the mitochondria to the nucleus in response to mitochondrial stress or growth factor stimulation in tumour cells. PDC catalyses pyruvate conversion to acetyl‐CoA in mitochondria and the nucleus.^4^ However, researchers have not determined whether PDC regulates histone acetylation.

Hydrogen sulphide (H_2_S), an important gasotransmitter in the cardiovascular system, is involved in vascular relaxation and antioxidant defences. Our previous study revealed that H_2_S sustained mitochondrial ATP production by regulating key enzymes in the mitochondrial tricarboxylic acid (TCA) cycle.^6^ Novel studies have shown that H_2_S covalently modifies cysteine residues on target proteins, through a process known as S‐sulfhydration. This modification modulates protein structure and activity.^7^, ^8^ As shown in our previous study, exogenous H_2_S inhibits VSMC proliferation under diabetic conditions.^9^ However, its mechanism remains unclear. The aim of the present study was to explore whether H_2_S regulates PDC translocation from the mitochondria to the nucleus to alter histone acetylation and subsequently inhibit VSMC proliferation.

## 2 MATERIALS AND METHODS

### 2.1 Animals

Leptin receptor‐deficient (db/db) mice (8‐10 weeks old, n = 60) and wild‐type C57BL/6 mice (n = 40) were purchased from the Animal Model Institute of Nanjing (Nanjing, China). The animals were housed under diurnal lighting conditions and provided standard mouse chow and water throughout the study period. Half of the db/db mice were allocated to the NaHS treatment group and intraperitoneally injected with NaHS (80 μmol/kg, Sigma) every 2 days for 12 weeks. All animal experiments were performed according to the Guide for the Care and Use of Laboratory Animals published by the China National Institute of Health and approved by the Animal Care Committees of Harbin Medical University, China.

### 2.2 Cell culture and treatments

Vascular smooth muscle cells from mouse aortae (VSMCs) were purchased from the Chinese Academy of Sciences Cell Bank. The media for cell lines was supplemented with 10% calf serum, 100 units/mL penicillin and 100 µg/mL streptomycin. VSMCs were maintained at 37℃ in a humidified incubator containing 5% CO_2_. Two days after seeding, the cultured VSMCs were randomly divided into the following treatment groups: control group (glucose, 25 mmol/L), high glucose (HG, 40 mmol/L) + palmitate (Pal, 500 µmol/L), HG+Pal+NaHS (100 µmol/L), HG+Pal+PPG (10 nmol/L, an inhibitor of CSE), HG+Pal+MitoTempo (2 µmol/L), HG+Pal+NAC (100 µmol/L), HG+Pal+NaHS+ACLI (50 µmol/L SB204990, an inhibitor of ATP citrate lyase) and HG+Pal+NaHS+PDHI (100 µmol/L CPI‐613, an inhibitor of PDC‐E1). Drugs were added to the culture medium for 24 hours. VSMCs treated with high glucose and palmitate classically mimic hyperglycaemia and hyperlipidaemia, respectively.

### 2.3 Cell count

Treated cells were prepared as a suspension that was slowly dripped onto the edge of the counting plate to fill the gap between the counting plate and the cover slip. After cells had spread on the counting plate, cells were observed and counted under a low power lens (10 × 10 times).

### 2.4 Functional nuclear isolation

Nuclei were isolated with the nuclei isolation kit: nuclei PURE prep from Sigma‐Aldrich. Briefly, VSMCs were washed with PBS and scraped from the plate in the presence of lysis buffer. VSMCs were carefully placed on top of a 1.8 mol/L sucrose gradient, and the resulting suspension was centrifuged at 30 000 *g* for 45 minutes in a precooled swinging bucket ultracentrifuge. Nuclei were collected as a white pellet at the bottom of the centrifuge tube and washed with nuclei storage buffer (provided with the kit). The purity of nuclei was assessed using immunoblotting. Isolated nuclei were used immediately in functional experiments.

### 2.5 Mitochondria isolation

Vascular smooth muscle cells were washed twice with ice‐cold PBS, resuspended in lysis buffer (mmol/L: 20 Hepes/KOH, pH 7.5, 10 KCl, 1.5 MgCl_2_, 1.0 sodium EDTA, 1.0 sodium EGTA, 1.0 dithiothreitol, 0.1 PMSF and 250 sucrose) and then homogenized with a homogenizer in an ice/water bath. After removing the nuclei and cell debris by centrifugation at 1000 *g* for 10 minutes at 4℃, the supernatants were subsequently centrifuged at 10 000 *g* for 10 minutes at 4℃. The resulting mitochondrial pellets were resuspended in lysis buffer. The supernatants and mitochondrial fractions were stored at −80℃.

### 2.6 Detection of H_2_S in VSMCs using the H_2_S probe 7‐azido‐4‐methylcoumarin

The fluorescence reaction of the sulphate diester in VSMCs was tested using 7‐azido‐4 methylcoumarin (C‐7Az, Sigma), which has been shown to selectively respond to H_2_S.^10^ VSMCs were incubated with 50 μmol/L C‐7Az PBS for 30 minutes and then washed with PBS. The fluorescence response of C‐7Az in VSMCs was detected using a fluorescence microscope (Olympus, XSZ‐D2) after excitation with a 720 nm laser. The results indicated that excitation fluorescence imaging was useful for detecting H_2_S by triggering the fluorescence reaction of C‐7Az.

### 2.7 Flow cytometry analysis of the cell cycle

Cells in the logarithmic growth phase were inoculated in 24‐well plates with 1 mL of media or in 6‐well plates at a density of 1 × 10^6^ cells/mL with 2 mL of media. The required treatment was performed (such as adding HG+Pal or NaHS), the culture was stopped at a specific time‐point, and the next experiment was performed. Cells were centrifuged at 68 *g* for 5 minutes, the supernatant was discarded, and the cell pellet was collected, washed twice with pre‐chilled PBS and fixed with pre‐chilled 70% ethanol at 4℃ for more than 4 hours. The sample was centrifuged at 239 *g* for 5 minutes, and the supernatant was discarded. The cell pellet was washed once with 3 mL of PBS and then incubated with 400 μL of CCAA solution (PI stain solution, green) and 100 μL of RNase A (100 μg/mL) at 4℃ in the dark for 30 minutes. Samples were detected using a flow cytometer with standard procedures, and generally 20 000 to 30 000 cells were counted. The results were analysed using the cell cycle fitting software ModFit.

### 2.8 Immunofluorescence staining

Vascular smooth muscle cells were fixed with 4% paraformaldehyde for 30 minutes and then permeabilized with 0.5% Triton X‐100 for 30 minutes. Cells on the coverslips were blocked with 5% BSA for 1 hour at 37℃, incubated with anti‐PDC‐E1 antibodies overnight at 4℃ and incubated with anti‐rabbit IgG for 1 hour. Analyses and photomicrography were performed using a fluorescence microscope.

### 2.9 Immunoprecipitation

Briefly, isolated mitochondria and nuclei were resuspended in PBS and diluted to a concentration of 1 mg/mL. After three freeze‐thaw cycles, 500 μg of protein from each sample was used for immunoprecipitation. Sepharose beads were conjugated with the anti‐HSP70 antibody (10 μg of antibody/500 μg of protein) and incubated with samples overnight at 4℃ with gentle rotation. Following the collection of beads using a centrifuge and three washing steps, precipitates were subjected to Western blot analyses to detect potential interacting proteins.

### 2.10 Analysis of mitochondrial and cellular ROS levels

Mitochondrial ROS and cellular ROS levels were analysed. Mitochondrial ROS production was measured using the MitoSOX Red mitochondrial superoxide indicator (Invitrogen). VSMCs were treated with control, HG, NaHS and MitoTempo for 24 hours. Cells were loaded with 5 μmol/L MitoSOX Red at 37℃ for 30 minutes. Red fluorescence was measured at 583 nm following excitation at 488 nm using a fluorescence microscope. Intracellular ROS levels were examined using the DCFH‐DA staining method based on the conversion of non‐fluorescent DCFH‐DA to highly fluorescent DCF upon intracellular oxidation by ROS. VSMCs were seeded on coverslips and incubated (45 minutes, 37℃, in the dark) in serum‐free media containing DCFH‐DA (10 μmol/L) in the presence of control, HG, NaHS and NAC. After the incubation, the conversion of DCFH‐DA to the fluorescent product DCF was measured using a spectrofluorometer with excitation at 484 nm and emission at 530 nm. Background fluorescence (conversion of DCFH‐DA in the absence of cells) was corrected by including parallel blanks.

### 2.11 Western blotting analysis

Cytoplasmic and nuclear fractions from all samples were quantified using the BCA Protein Assay kit (Beyotime), separated by electrophoresis on SDS‐polyacrylamide gels and transferred to nitrocellulose membranes. The antibodies used for Western blot analyses included anti‐CSE (42 kD, 1:1000), anti‐CBS (61 kD, 1:1000), anti‐β‐actin (42 kD, 1:1000), anti‐MMP2 (72 kD, 1:1000), anti‐MMP9 (67‐92 kD, 1:1000), anti‐OPN (66 kD, 1:1000), anti‐α‐smooth muscle actin (42 kD, 1:1000), anti‐cyclin D1 (36 kD, 1:1000), anti‐PCNA (36 kD, 1:1000), anti‐PDC‐E1 (43 kD, 1:1000), anti‐H3 (17 kD, 1:1000), anti‐H3K9 (17 kD, 1:1000), anti‐H3K18 (17 kD, 1:1000), anti‐SOD (15 kD, 1:1000), anti‐CAT (55 kD, 1:1000), anti‐HSP70 (70 kDa, 1:1000), anti‐COX IV (16 kD, 1:1000) and anti‐Lamin B1 (66 kD, 1:1000) antibodies. All antibodies were purchased from Proteintech Group, Inc. Protein‐antibody complexes were incubated for 1 hour at room temperature with an anti‐mouse/anti‐rabbit antibody. Densitometry was conducted with the image processing and analysis program AlphaView SA, and the data are reported as relative units.

### 2.12 Point mutation of PDC‐E1

Adenoviruses expressing GFP and PDC‐E1‐GFP were purchased from Cyagen Biosciences Inc. The 101 cysteine site of PDC‐E1(PDHA1) was mutated to alanine. With the method of gateway molecular technology, we cloned the synthesized DNA fragment into pDONR vector (Invitrogen) via BP reaction to get Entry clone which further recombinated with the destination vector (pAV[Exp]‐CMV>shuttle empty vector) via LR reactions to obtain the final expression clones (pAV[Exp]‐CMV>{mPDHA1(mutant)}/FLAG:IRES:EGFP). The positive clones at each step were screened by DNA sequencing. The adenovirus was added directly to cells, and after 24 hours of transfection, new, fresh medium was added. The cells received different treatments 24 hours after transfection, and the related proteins were detected using Western blotting.

### 2.13 Pyruvate dehydrogenase activity assay

The PDH activity in the nuclear lysates was measured with a colorimetric PDH activity assay kit (GENMED) according to the manufacturer's instructions, and the absorbance at 450 nm was measured kinetically for approximately 30 minutes at 37℃ after the addition of PDH developer and PDH substrate. Values for the blank sample (without PDH substrate) were subtracted from the sample readings, and PDH activity (nmol NADH/min) was normalized to mg of protein.

### 2.14 Acetyl‐coenzyme A analysis

Acetyl‐coenzyme A was measured according to the manufacturer's protocol (acetyl‐coenzyme A assay kit, Sigma). All samples and standards were analysed in duplicate. Ten microlitres of each sample was added to duplicate wells of a 96‐well plate. Samples were brought to a final volume of 50 μL with acetyl‐CoA assay buffer. A blank sample for each sample was prepared by omitting the Conversion Enzyme in the Reaction Mix. Ten microlitres of acetyl‐CoA quencher was added to each sample, standard and sample blank well to correct for the background signal created by free coenzyme A and succinyl‐CoA. Plates were incubated at room temperature for 5 minutes. Two microlitres of Quench Remover was added to each well, mixed well and incubated for an additional 5 minutes. The plate was mixed well using a horizontal shaker or by pipetting, and the reaction proceeded for 10 minutes at 37℃ in the dark. The fluorescence intensity was subsequently measured (λex = 535/λem = 587 nm).

### 2.15 S‐sulfhydration assay

S‐sulfhydration was performed using a previously described method.^7^ VSMCs Were homogenized in HEN buffer solution containing 250 mmol/L HEPES‐NaOH, 1 mmol/L EDTA, 0.1 mmol/L neocuproine and 100 mmol/L deferoxamine with the pH adjusted to 7.7. The cell lysate was prepared using HEN buffer containing 0.5% CHAPS, 0.1% SDS, 20 mmol/L methyl methanethiosulphonate, 10 μg/mL leupeptin, 5 μg/mL aprotinin and 1 mmol/L protease inhibitor PMSF. Quantitative analyses were performed on cell lysates. VSMCs were added to blocking buffer (HEN buffer containing 2.5% SDS and 20 mmol/L methyl methanethiosulphonate) and incubated at 50℃ for 60 minutes with frequent vortexing. Four volumes of cold acetone were added to each 15‐mL centrifuge tube and incubated at −20℃ for 1 hour. Then, the tubes were centrifuged at 2000 *g* for 10 minutes at 4℃ and the cold acetone was removed. The cells were resuspended in 90 μL of HEN buffer (containing 1% SDS) and transferred to a new 1.5‐mL EP tube. Then, 4 mmol/L N‐[6‐(biotinamido) hexyl]‐3‐(2‐pyridyldithio) propionamide (biotin‐HPDP stop solution) was added and incubated at 25℃ for 1 hour. After an incubation for 3 hours at 25℃, biotinylated proteins were precipitated with streptavidin‐agarose beads, which were subsequently washed with HEN buffer. The biotinylated proteins were eluted by SDS‐PAGE and subjected to Western blot analysis using antibodies against PDC‐E1.

### 2.16 Measurement of intracellular polysulphide levels

Intracellular production of polysulphide was monitored using a newly developed fluorescent probe, SSP4, with slight modifications.^11^ Briefly, VSMCs were loaded with 50 μmol/L SSP4 in serum‐free DMEM containing 0.003% Cremophor EL for 15 minutes at 37℃ in the dark. After washing, SSP4 was detected using a fluorescence microscope (Olympus, XSZ‐D2).

### 2.17 Statistical analysis

Statistical analyses of the data were performed using GraphPad Prism software. Two groups were compared using unpaired Student's *t* tests, and multiple groups were compared using one‐way ANOVA followed by multiple comparison tests. Statistical significance was detected at the *P* < .05 level. The results are presented as the means ± SEM of multiple experiments.

## 3 RESULTS

### 3.1 CSE expression and H_2_S levels in vascular smooth muscle cells under diabetic conditions

**FIGURE 1:**
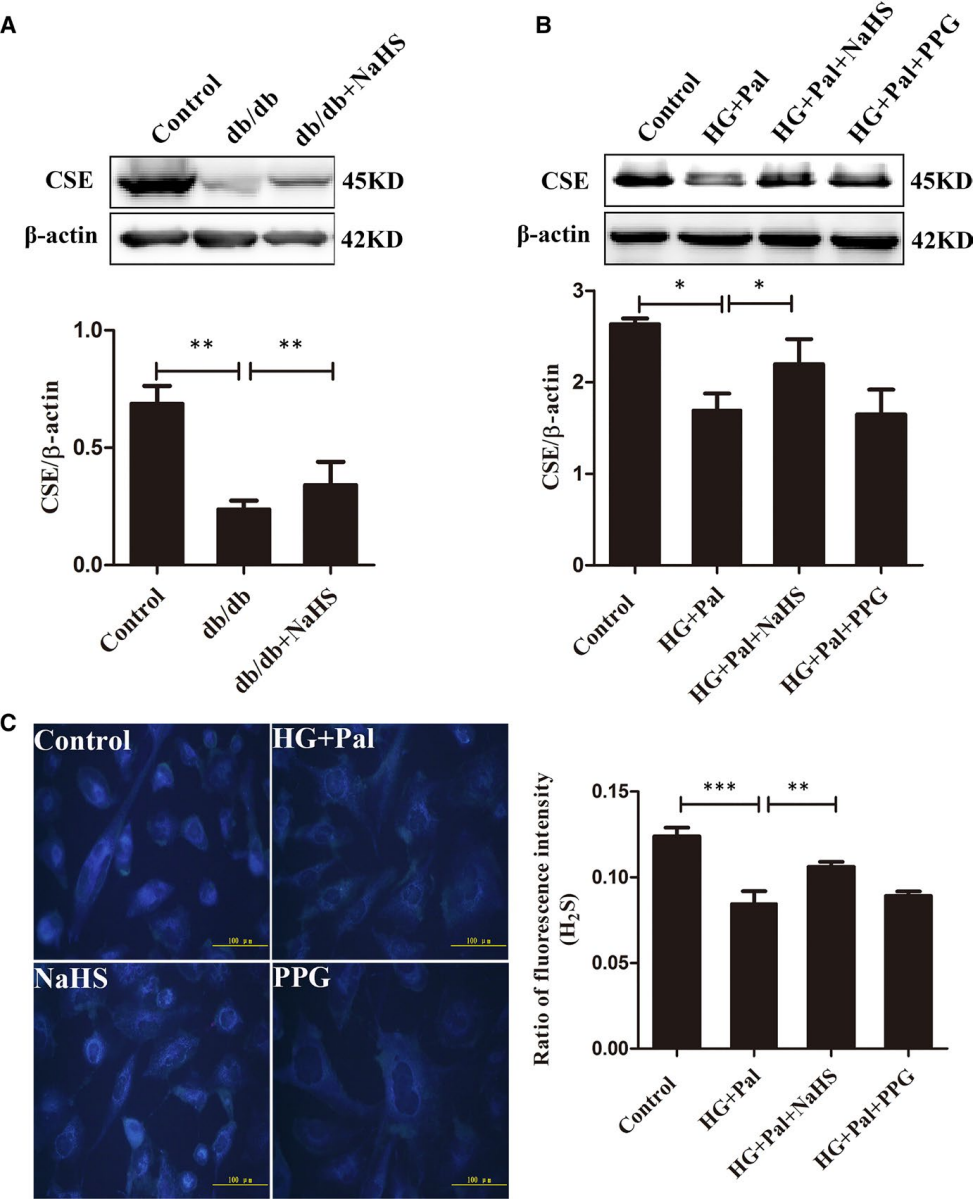
Exogenous H_2_S alters CSE expression and the H_2_S content. A, CSE expression in tissues was quantified by performing Western blotting (n = 4). B, vascular smooth muscle cells (VSMCs) were treated with HG (40 mmol/L) + palmitate (Pal, 500 μmol/L), HG+Pal+NaHS (100 μmol/L) or HG+Pal+PPG (10 nmol/L, an irreversible competitive CSE inhibitor) for 24 h. The CSE protein expression level was determined using Western blotting (n = 5). C, Staining with the 7‐azido‐4‐methylcoumarin probe was used to measure H_2_S levels in VSMCs (n = 4). Values are presented as the means ± SD *P < .05, **P < .01 and ***P < .001

In mammalian cells, the generation of H_2_S mainly depends on cystathionine‐γ‐lyase (CSE) and cystathionine‐β‐synthase (CBS). A tissue‐specific distribution of the two enzymes required for H_2_S production has been observed. CBS is the main enzyme required for H_2_S generation in the nervous system. CSE is the predominant enzyme for H_2_S production in the cardiovascular system. However, a recent study found that CBS also generates H_2_S in the cardiovascular system.^12^ As shown in our previous study, the expression of CSE, an enzyme that generates H_2_S, and H_2_S levels was decreased in the mesenteric artery of animals with STZ‐induced type 1 diabetes.^13^ In this study, we chose db/db mice as the animal model of type 2 diabetes. The expression of CSE in the thoracic artery was detected. A lower level of the CSE protein was detected in db/db mice than in control mice. CSE protein levels were restored by the NaHS treatment in mice (db/db+NaHS) (Figure 1A). The expression of CBS was similar among the different groups (Figure S1).

Vascular smooth muscle cells from mouse aortae were cultured with high glucose (HG, 40 mmol/L) and palmitate (Pal, 500 µmol/L) for 24 hours to mimic the hyperglycaemia and hyperlipidaemia, respectively, observed in individuals with type 2 diabetes. Similarly, CSE expression was significantly decreased by HG and Pal treatments, while exogenous H_2_S restored its expression. DL‐propargylglycine (PPG, 10 nmol/L) is an inhibitor of CSE. The PPG treatment decreased the level of the CSE protein (Figure 1B). We tested the ubiquitylation level of CSE with immunoprecipitation to explain the decrease in levels of the CSE protein in the HG+Pal group. MG132 is an inhibitor of the 26S proteasome. The level of CSE ubiquitylation was significantly increased in the HG+Pal group compared to the control and NaHS‐treated and MG132 groups (Figure S2). The H_2_S fluorescence probe 7‐azido‐4‐methylcoumarin (C‐7Az) was used to measure the H_2_S content in VSMCs. The H_2_S levels in the HG+Pal and PPG groups were significantly lower than those in the control and exogenous H_2_S groups (Figure 1C). These results indicated decreased endogenous H_2_S production in VSMCs due to CSE degradation under hyperglycaemic and hyperlipidaemic conditions.

### 3.2 Effects of H_2_S on the proliferation and migration of VSMCs stimulated with high glucose and palmitate

**FIGURE 2:**
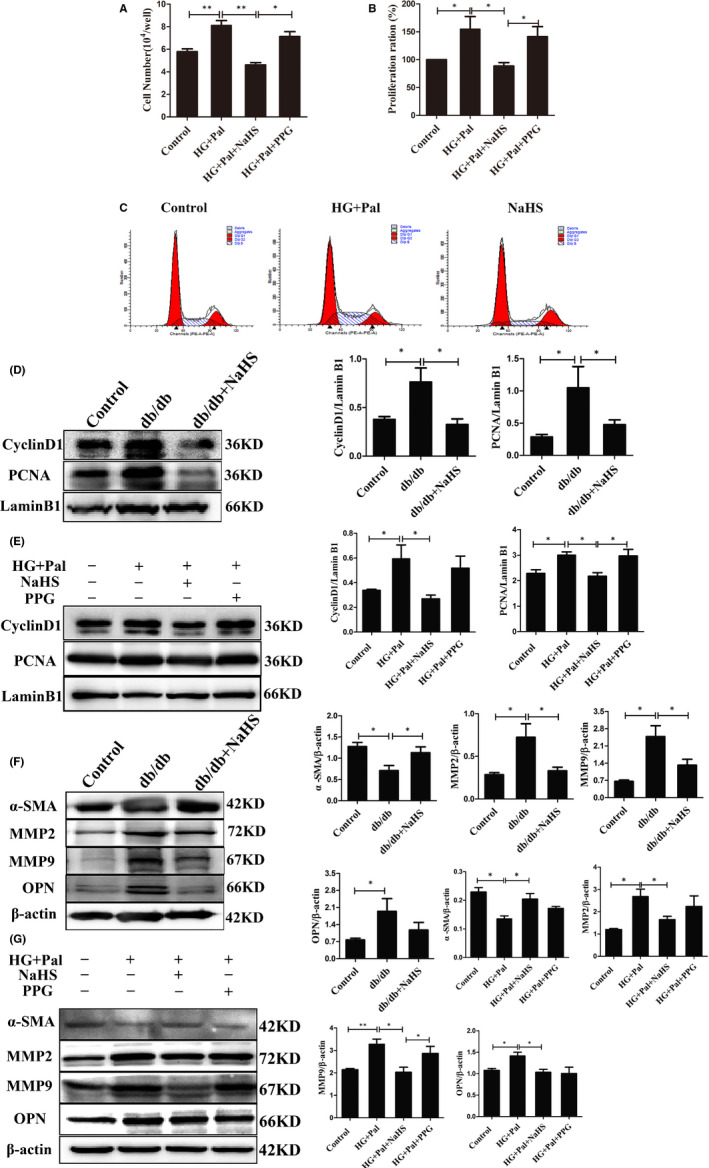
Effects of exogenous H_2_S on proliferation and phenotypic changes. A, vascular smooth muscle cells (VSMCs) were treated with HG (40 mmol/L) + palmitate (Pal, 500 μmol/L), HG+Pal+NaHS (100 μmol/L) or HG+Pal+PPG (10 nmol/L, an irreversible competitive CSE inhibitor). Cell counting was performed to detect the number of cells after 24 h of treatment (n = 4). B, CCK‐8 assays were used to detect the effects of different drug treatments on cell viability (n = 6). C, Flow cytometry assay of the cell cycle of VSMCs (n = 3). D, PCNA and CyclinD1 expression levels in aortas from control mice, db/db mice and db/db mice treated with NaHS (n = 4). E, PCNA and CyclinD1 expression levels in VSMCs treated with HG (40 mmol/L) + palmitate (Pal, 500 μmol/L), HG+Pal+NaHS (100 μmol/L), or HG+Pal+PPG (10 nmol/L, an irreversible competitive CSE inhibitor) (n = 5). (F and G) Expression levels of the α‐SMA, MMP2, MMP9 and OPN proteins in tissues (F) and VSMCs (G) were determined using western blot analyses (n = 4). Values are presented as the means ± SD. *P < .05, **P < .01 and ^#^ *P* < .05 compared with the HG+Pal group

We examined whether H_2_S levels regulated VSMC proliferation. Proliferation assays showed that high glucose and palmitate induced cell growth, whereas exogenous H_2_S blocked cell growth (Figure 2A, B). H_2_S significantly retarded cell progression at G1 phase, and the number of cells in S phase decreased (Figure 2C and Table 1). Next, the protein levels of cyclin D1, a G1/S checkpoint protein, and PCNA, which is responsible for governing the G1/S transition, were assessed. Levels of the cyclin D1 and PCNA proteins were significantly increased in the thoracic arteries of db/db mice and VSMCs treated with high glucose and palmitate compared to those treated with exogenous H_2_S (Figure 2D, E). Additionally, HG and palmitate significantly enhanced VSMC migration rates two‐ and threefold at 6, 12 and 24 hours, respectively, compared to the control groups using the scratch assay. PPG also increased VSMC migration rates. However, exogenous H_2_S inhibited VSMC migration (Figure S3).

**TABLE 1:** Exogenous H_2_S effected on vascular smooth muscle cell (VSMC) cell cycle

VSMCs	G1	S	G2
Control	60.81% ± 2.370%	22.50% ± 1.738%	16.69% ± 0.8171%
HG+Pal	44.56% ± 5.829%**	40.85% ± 4.060%**	14.59% ± 2.332%
HG+Pal+NaHS	53.74% ± 4.916%#	29.84% ± 9.369%#	16.43% ± 4.576%

** *P* < .01 vs Control.

# *P* < .05 vs HG+Pal (n = 3).

Hyperproliferative SMCs induce a phenotypic switch of VSMCs. The biomarkers α‐SMA, which represents the contractile type of SMCs, OPN and MMP2/9, which represent the synthetic phenotype, were examined. The expression of α‐SMA was significantly attenuated in db/db mice and HG‐ and palmitate‐stimulated VSMCs compared to cells treated with exogenous H_2_S. In contrast, the expression of OPN and MMP2/9 was significantly increased in the thoracic arteries of db/db mice and VSMCs treated with high glucose and palmitate. Pretreatment with PPG increased OPN, MMP2 and MMP9 expression (Figure 2F, G). Taken together, these results validated that the decrease in H_2_S levels promoted VSMC proliferation in the diabetic state.

### 3.3 H_2_S inhibits PDC‐E1 translocation from the mitochondria to the nucleus under high glucose and palmitate conditions

**FIGURE 3:**
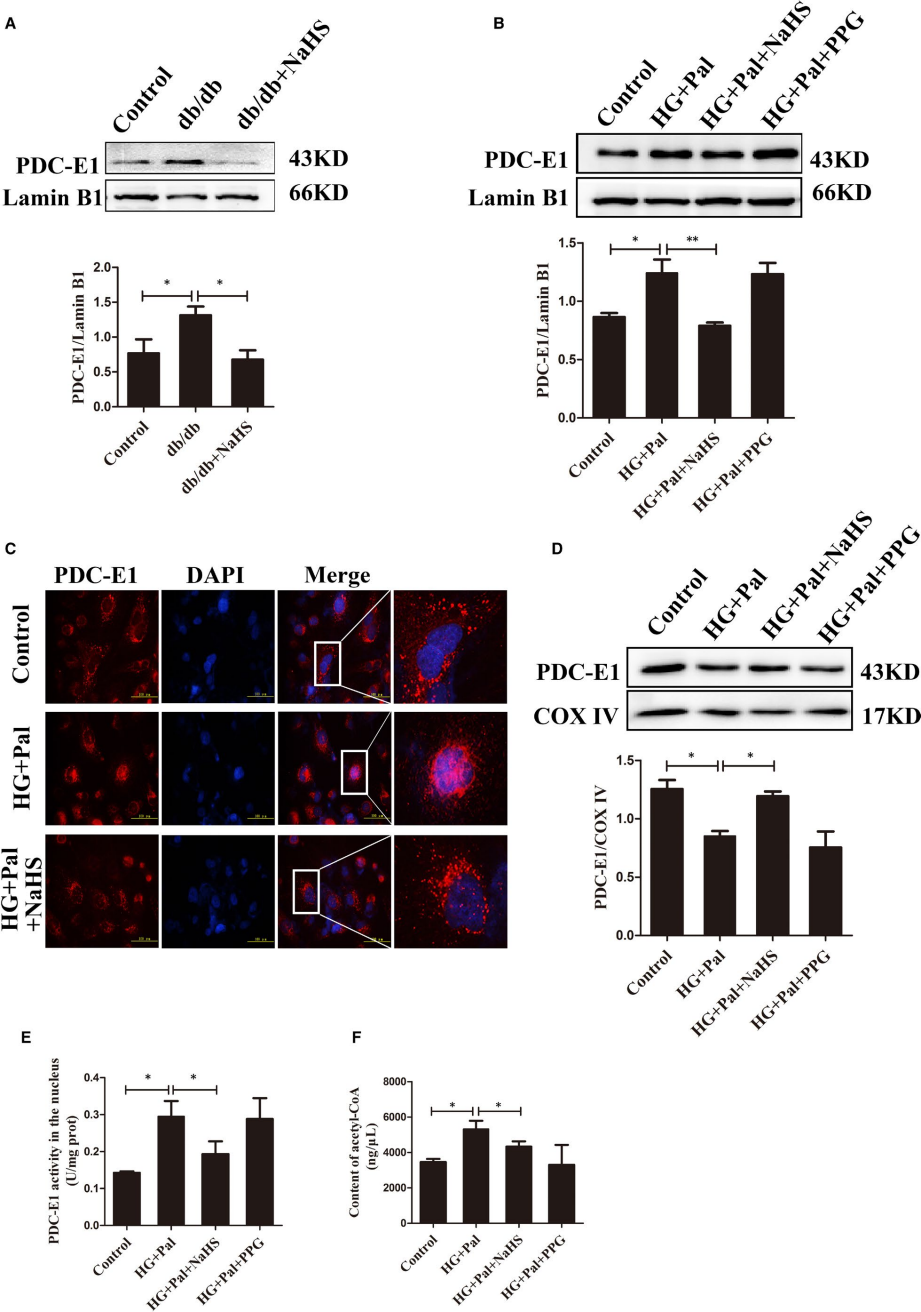
H_2_S inhibits pyruvate dehydrogenase complex‐E1 (PDC‐E1) translocation from the mitochondria to the nucleus of cells stimulated with high glucose and palmitate. A and B, Nuclei were extracted from tissues (A) and cells (B) separately to detect the level of the PDC‐E1 protein in the nucleus (n = 4). C, Immunofluorescence staining was used to detect the nuclear localization of PDC‐E1. D, The levels of PDC‐E1 in the mitochondria of VSMCs (n = 5). E, The activity of PDC‐E1 in the nuclei of VSMCs (n = 4). F, Detection of the acetyl‐CoA content in VSMCs (n = 4). Values are presented as the means ± SD *P < .05 and **P < .01

Pyruvate dehydrogenase complex is composed of three catalytic enzymes: pyruvate dihydrolipoamide (E1), dihydrolipoamide transacetylase (E2), and dihydrolipoamide dehydrogenase (E3). PDC‐E1 is responsible for catalysing the conversion of pyruvate to acetyl‐CoA. According to some studies, PDC‐E1 translocates from the mitochondria to the nucleus in response to mitochondrial stress.^14^ We first examined the nuclear level of PDC‐E1 in the thoracic aorta and VSMCs exposed to hyperglycaemia and hyperlipidaemia. We extracted nuclei from VSMCs, and the Western blot analysis showed a higher level of the PDC‐E1 protein in db/db mice and VSMCs stimulated with high glucose and palmitate than that in the control and exogenous H_2_S treatment groups (Figure 3A, B). We also observed that PDC‐E1 was evidently localized within the nucleus (marked with DAPI) upon the administration of high glucose and palmitate using immunofluorescence microscopy (Figure 3C). We extracted the mitochondria and then measured the level of the PDC‐E1 protein to further confirm that PDC‐E1 detected in the nucleus was derived from mitochondria. We observed a lower level of the PDC‐E1 protein in mitochondria from the HG+Pal group than in the control and NaHS groups (Figure 3D). These results suggested that high glucose and palmitate promoted the translocation of PDC‐E1 from the mitochondria to the nucleus; however, exogenous H_2_S reduced PDC‐E1 translocation.

We separated nuclei from VSMCs through high‐sucrose gradient centrifugation, avoiding mitochondrial contamination, to evaluate whether nuclear PDC‐E1 was functional. The activities of PDC‐E1 in nuclei were tested. The activity of PDC‐E1 in the HG+Pal group was significantly increased compared to the control and NaHS treatment groups (Figure 3E). We then detected the acetyl‐CoA concentration in the nuclei and detected obviously higher levels in the HG+Pal group than in the control and exogenous H_2_S groups (Figure 3F). Based on these results, PDC‐E1 translocation from the mitochondria to the nucleus after treatment with high glucose and palmitate was involved in acetyl‐CoA production.

### 3.4 Nuclear PDC‐E1 promotes histone acetylation and cell proliferation

**FIGURE 4:**
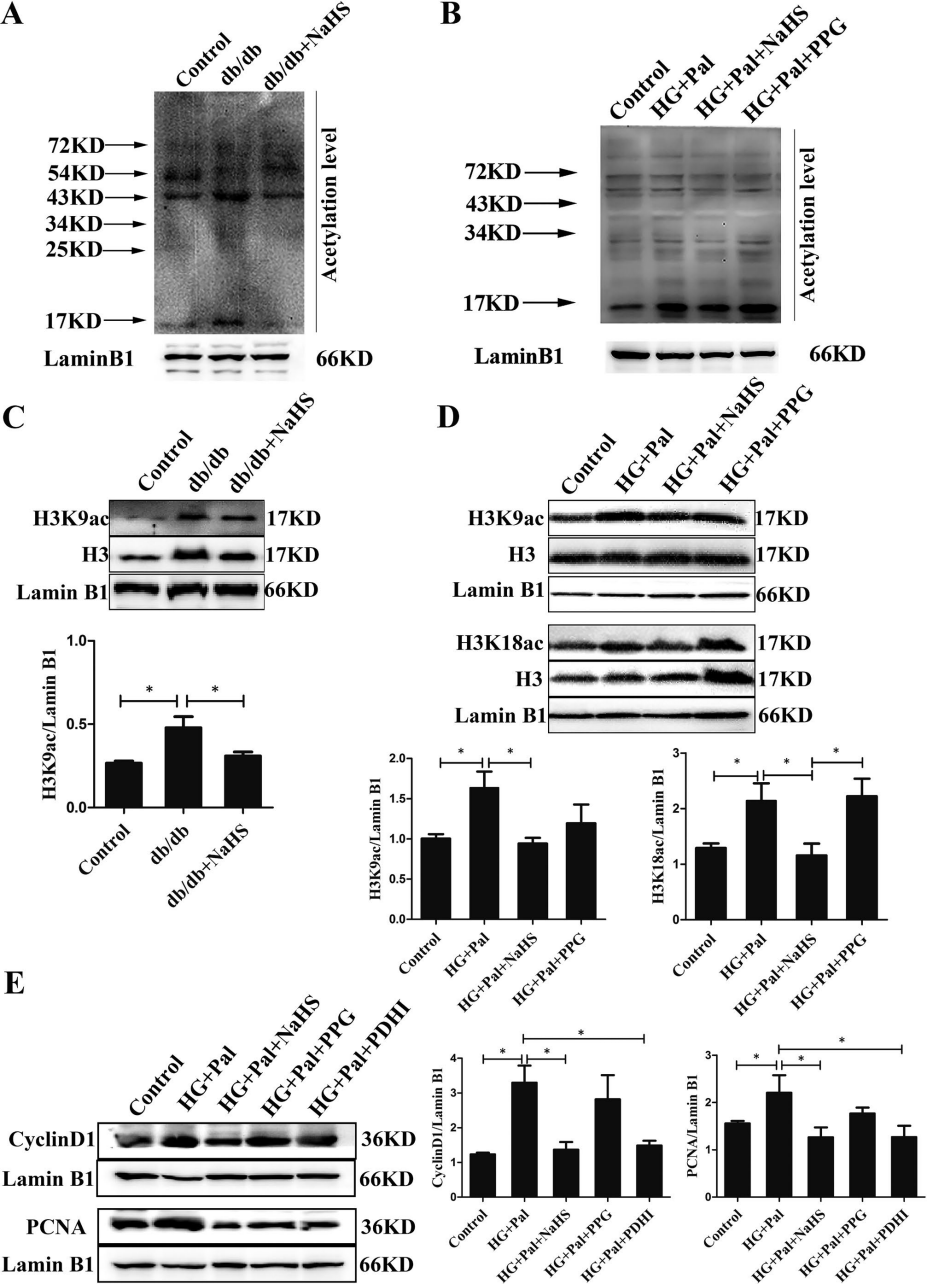
Nuclear pyruvate dehydrogenase complex‐E1 (PDC‐E1) promotes histone acetylation and cell proliferation. A and B, Relative acetylation levels in the nuclei of the thoracic aorta (A) and VSMCs (B). C, The levels of H3 and H3K9ac in nuclear extracts of the thoracic aorta (n = 4). D, The levels of H3, H3K9ac and H3K18ac in nuclear extracts of VSMCs (n = 4). E, PDC inhibitor (PDHI, 100 μmol/L) inhibited the proliferation of VSMCs (n = 3). Values are presented as the means ± SD *P < .05

We showed that nuclear PDC‐E1 generated acetyl‐CoA and then confirmed whether acetyl‐CoA modified histones. We first extracted the nuclei from the thoracic aorta and VSMCs and examined the acetylation level. The acetylation level in db/db mice and VSMCs treated with high glucose and palmitate was higher than that in the control and cells treated with NaHS (Figure 4A, B). Next, we detected the specific acetylation site of H3K9, which is involved in regulating gene expression in proliferating VSMCs. After exposure to high glucose and palmitate, the level of H3K9 acetylation was increased compared to that in the control and NaHS‐treated groups (Figure 4C, D). ATP citrate lyase (ACL) is an acetyl‐CoA‐generating enzyme localized in the cytosol and nuclei that provides an acetyl group for histone acetylation. We used ACLI (ACL inhibitor, SB204990, 50 μmol/L) and a PDC‐E1 inhibitor (PDHI, CPI‐613, 100 μmol/L) to study the relative importance of these two enzymes in nuclear acetylation. PDHI (Figure 4E) and ACLI (Figure S4) obviously reduced the expression of cyclin D1 and PCNA in VSMC nuclei under diabetic conditions. Therefore, exogenous H_2_S decreased the level of H3K9 acetylation by inhibiting PDC‐E1 translocation from the mitochondria to the nucleus.

### 3.5 mtHSP70 assists with the nuclear translocation of PDC‐E1 from mitochondria

**FIGURE 5:**
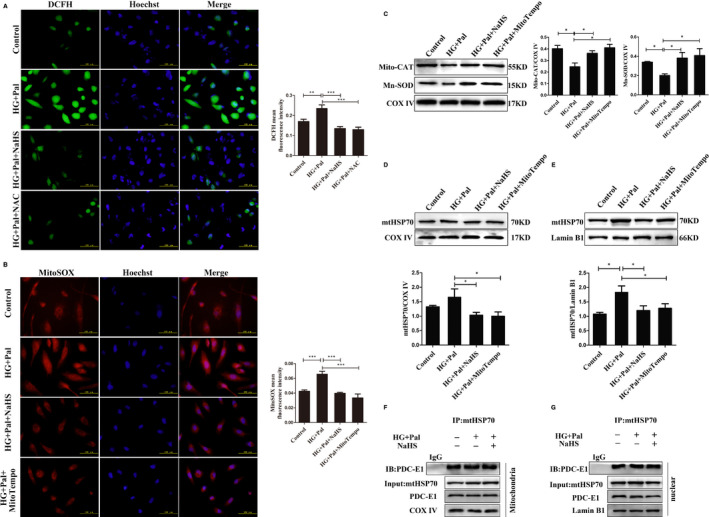
Exogenous H_2_S inhibits oxidative stress in vascular smooth muscle cells (VSMCs). A, The total ROS level was measured using DCFH (green fluorescence), and B, mitochondrial ROS level was measured using MitoSOX (red fluorescence). The mean fluorescence intensity was measured. C, Mitochondrial Mito‐CAT and Mn‐SOD levels were examined using western blotting (n = 4). D, The mitochondria were extracted from VSMCs, and the expression of the mtHSP70 protein in each group was detected using western blotting (n = 4). E, Nuclear proteins were extracted from VSMCs, and the expression of the mtHSP70 protein in each group was detected using western blotting (n = 4). F, The mitochondrial extracts from VSMCs were immunoprecipitated with an anti‐mtHSP70 antibody and then immunoblotted with antibodies specific for PDC‐E1. G, The nuclear extracts from VSMCs were immunoprecipitated with an anti‐mtHSP70 antibody and then immunoblotted with antibodies specific for PDC‐E1. Values are presented as the means ± SD. *P < .05

Mitochondria adapt to stress induced by increased ROS levels, which leads to increased expression of heat shock proteins and mitochondrial transporters, promoting communication with the nucleus.^15^ We examined whether high glucose and palmitate induced oxidative stress. We measured intracellular and mitochondrial reactive oxygen species (ROS) levels with the fluorescent probes DCFH and MitoSOX. The quantification of DCFH and MitoSOX fluorescence intensities showed similar increases following the administration of high glucose and palmitate, while exogenous NaHS, NAC and MitoTempo significantly attenuated intracellular ROS levels and mitochondria (Figure 5A, B). MitoTempo is a suppressor of ROS production in mitochondria. The expression of mitochondrial catalase (Mito‐CAT) and manganese‐dependent superoxide dismutase (Mn‐SOD) was measured to further examine the role of exogenous H_2_S in ROS production in VSCMs treated with HG and palmitate. The expression of Mito‐CAT and Mn‐SOD, which was downregulated in the HG+Pal group, was upregulated by the NaHS treatment (Figure 5C). Our data reinforced the concept that exogenous H_2_S attenuated oxidative stress in VSMCs.

Next, we investigated whether mitochondrial chaperones are involved in PDC‐E1 translocation. We chose mtHSP70 (mitochondrial heat shock protein 70) based on previous studies showing that HSP70 participates in the nuclear transport of several mitochondrial proteins.^16^, ^17^ We extracted mitochondria, and Western blot assays revealed a significantly higher level of the mtHSP70 protein in the HG+pal group than in the control, exogenous H_2_S and MitoTempo groups (Figure 5D). We also confirmed a noticeable increase in the level of nuclear mtHSP70 in the HG+pal group (Figure 5E). We performed immunoprecipitation to detect the interaction between mtHSP70 and PDC‐E1. Our results suggested that mtHSP70 may bind to PDC‐E1 in the nucleus and mitochondria (Figure 5F, G). Based on these results, mtHSP70 assisted with PDC‐E1 translocation induced by mitochondrial oxidative stress.

### 3.6 Exogenous H_2_S regulates PDC‐E1 S‐sulfhydration to inhibit VSMC proliferation

**FIGURE 6:**
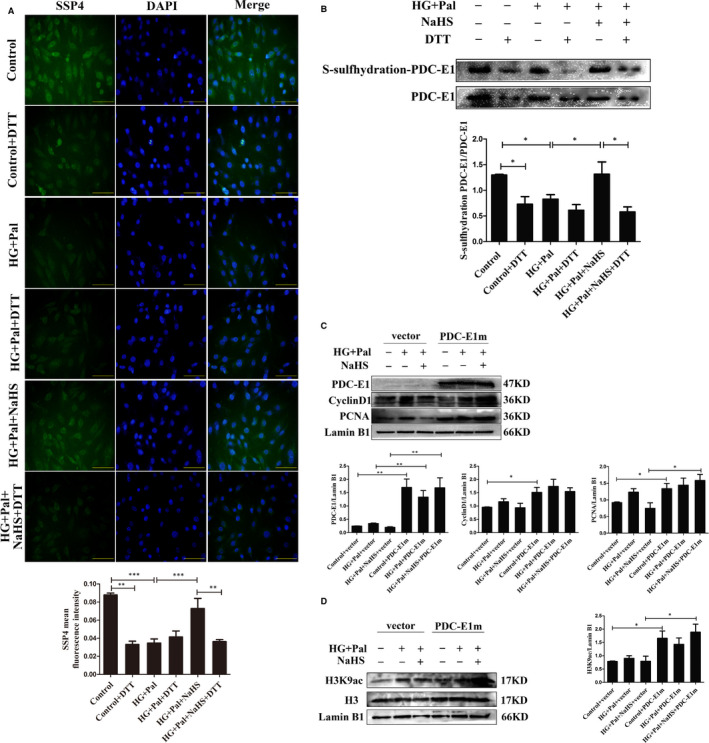
Exogenous H_2_S regulates pyruvate dehydrogenase complex‐E1 (PDC‐E1) S‐sulfhydration to promote PDC‐E1 translocation. A, The polysulphidation level was measured with a fluorescent probe, SSP4, in vascular smooth muscle cells. Scale bar = 100 μm. Dithiothreitol (1 mmol/L, 30 min, an inhibitor of disulphide bond formation). B, Quantification of polysulfidation in VSMCs was performed using western blotting (n = 4). C, The cysteine 101 to alanine PDC‐E1 mutant (PDC‐E1m) and the vector control were transfected into VSMCs for 6 h and then cells were treated with HG (40 mmol/L) or palmitate (Pal, 500 μmol/L) in the presence or absence of NaHS (100 μmol/L) for 24 h. The expression levels of PDC‐E1, CyclinD1 and PCNA were detected using western blotting (n = 5). D, The levels of H3 and H3K9ac were detected using western blotting after the transfection of the mutant (n = 5). Values are presented as the means ± SD. *P < .05, **P < .01 and ***P < .001

**FIGURE 7:**
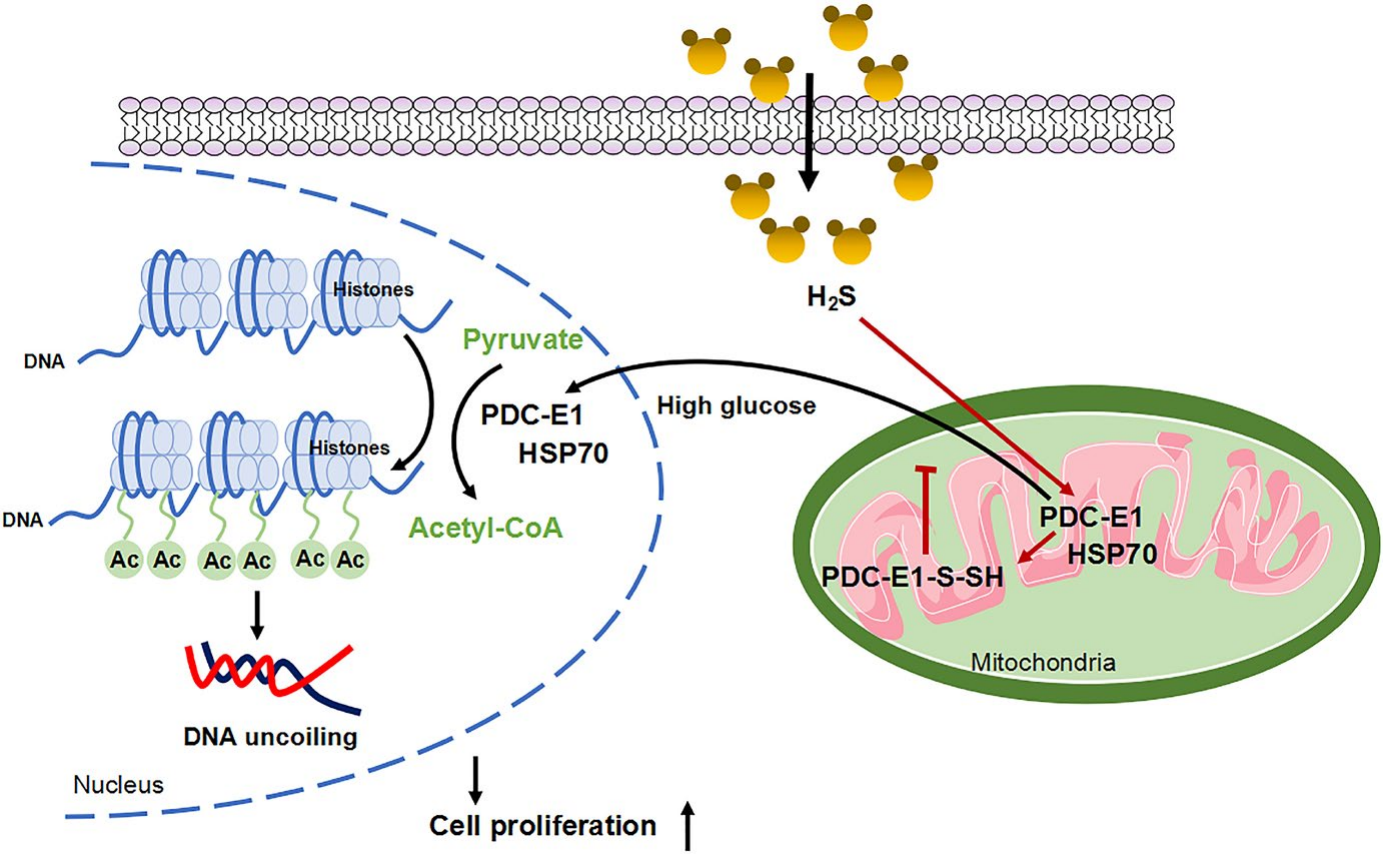
Exogenous H_2_S regulates pyruvate dehydrogenase complex‐E1 (PDC‐E1) translocation from the mitochondria to the nucleus

As shown in Figure 3E, exogenous H_2_S decreased the activity of PDC‐E1. We detected PDC‐E1 S‐sulfhydration to further study how exogenous H_2_S inhibits PDC‐E1 activity. The H_2_S modification of specific cysteine residues of proteins, which is referred to as S‐sulfhydration, has been extensively studied. Protein S‐sulfhydration, a post‐translational modification, is involved in alterations in protein structure and activity. SSP4, a fluorescent probe, was used to detect S‐sulfhydration. S‐sulfhydration was obviously observed after treatment with exogenous H_2_S. Dithiothreitol (DTT, 1 mmol/L), an inhibitor of S‐sulfhydration, reduced the effect of NaHS on S‐sulfhydration (Figure 6A). Furthermore, a biotin‐switch assay was also used to measure S‐sulfhydration in proteins. NaHS evidently increased the S‐sulfhydration of PDC‐E1, whereas DTT abolished the S‐sulfhydration of PDC‐E1 (Figure 6B). We used bioinformatics methods to analyse and predict the structure of the active centre of PDC‐E1. PDC‐E1 consists of 390 amino acids and contains 11 cysteine residues. Based on the bioinformatics analysis, Cys101 of PDC‐E1 in the active centre was mutated to alanine (Figure S5) and a mutant PDC‐E1‐Cys101 (PDC‐E1m) overexpression plasmid was constructed. After PDC‐E1m overexpression, H_2_S did not reduce the expression of cyclin D1 and PCNA in the high glucose and palmitate groups or the exogenous H_2_S treatment group (Figure 6C). Moreover, after PDC‐E1m overexpression, the level of H3K9 acetylation was increased in the HG+Pal and exogenous H_2_S groups (Figure 6D). Taken together, H_2_S inhibited VSMC proliferation by increasing PDC‐E1 S‐sulfhydration and reducing PDC‐E1 translocation (Figure 7).

## 4 DISCUSSION

In our study, PDC‐E1 was present and functional in the nucleus in VSMCs stimulated with high glucose and palmitate. Nuclear PDC‐E1 produced acetyl‐CoA that was used for histone acetylation to promote VSMC proliferation. Exogenous H_2_S inhibited PDC‐E1 translocation from the mitochondria to the nucleus via PDC‐E1 S‐sulfhydration to decrease VSMC proliferation.

Based on accumulating evidence, VSMC proliferation is a characteristic of patients with type 2 diabetes. When VSMC proliferation occurs, VSMCs switch from a contractile phenotype to a synthetic phenotype and cell cycle–associated genes are overexpressed. In our study, the expression of PCNA and cyclin D1 was significantly increased in cells stimulated with high glucose and palmitate. Some studies have revealed that stress reactions, such as oxygen deficiency or a high metabolic state, induce alterations in the post‐translational modifications (PTMs) of histones.^18^, ^19^ PTMs have been considered key contributors to controlling target gene expression under both physiological and pathophysiological conditions. For example, histone acetylation by histone acetyltransferases contributes to active transcription by rendering gene promoters more accessible to the transcriptional machinery.^20^ Evidence has documented the emergence of metabolic enzymes, such as ATP citrate lyase and PDC‐E1, as crucial modulators of cell proliferation. PDC‐E1 is a mitochondrial complex that functions as a gatekeeper to regulate pyruvate flux from the cytosol to mitochondria, which couples glycolysis to OXPHOS.^21^ Relocalization of PDC‐E1 has been observed in cancer cells that were serum‐starved or stimulated with epidermal growth factor or a mitochondrial respiration inhibitor (rotenone).^22^ PDC‐E1 converts mitochondrial pyruvate into acetyl‐CoA. Thus, the concentration of acetyl‐CoA in mitochondria may be 20‐ to 30‐fold greater than that in the cytoplasm and nucleus because acetyl‐CoA is membrane‐impermeable.^23^ Therefore, PDC‐E1 must be translocated to the nucleus from mitochondria to generate acetyl‐CoA. We confirmed that mitochondrial oxidative stress promoted PDC‐E1 translocation to the nucleus. In our study, the activity of PDC‐E1 and the acetyl‐CoA content in the nucleus increased in the HG+Pal group. Exogenous H_2_S reduced PDC‐E1 activity and the acetyl‐CoA content in the nucleus. H3K9 acetylation levels were significantly increased under hyperglycaemic and hyperlipidaemic conditions due to PDC‐E1 translocation; however, exogenous H_2_S reduced H3K9 acetylation. Thus, acetyl‐CoA, an important substrate for histone acetylation, is generated in the nucleus from pyruvate through a mechanism that depends on PDC‐E1 translocation to the nucleus.

Hydrogen sulphide plays a crucial role in the physiology and pathophysiology of the cardiovascular system. In our study, we noticed that the expression of CSE, a key enzyme to produce H_2_S in cardiovascular system, was decreased in db/db mice (Figure 1A, B). Immunoprecipitating to detect the interaction between CSE and ubiquitin, We found CSE was modified by ubiquitination and degraded by ubiquitin‐proteasome system, thus reduced the expression level of CSE. Exogenous H_2_S alleviated the ubiquitination and degradation of CSE, thereby increasing the expression of CSE (Figure S2). The persulphidation or S‐sulfhydration of reactive cysteines (ie Cys‐SSH) has recently been shown to contribute to cellular redox homeostasis. Persulphidation or S‐sulfhydration is generated by the transsulphuration pathway, which catabolizes cysteine and cystathionine to generate hydrogen sulphide (H_2_S) and H_2_S‐related sulphane sulphur compounds (referred to as H_2_Sn).^24^, ^25^ This pathway is of particular importance for the vascular system because it expresses CSE.^26^ Based on emerging data, the hydropersulphide moiety (‐SSH) in the active cysteine residues of target proteins mediates cellular functions. In our study, exogenous H_2_S modified PDC‐E1 via S‐sulfhydration, which inhibited its activity and nuclear translocation. The administration of DTT, a blocker of S‐sulfhydration, decreased the S‐sulfhydration level of PDC‐E1. We overexpressed PDC‐E1 in which Cys101 was mutated to Ala and showed that treatment with exogenous H_2_S decreased the PDC‐E1 S‐sulfhydration level and increased PDC‐E1 translocation to the nucleus. Thus, S‐sulfhydration of PDC‐E1 at Cys101 might be one of the specific mechanisms by which H_2_S reduced PDC‐E1 translocation to the nucleus and histone acetylation and inhibited VSMC proliferation (Figure 7).

In this study, PDC‐E1, a key mitochondrial TCA cycle‐related enzyme, translocated to the nucleus, and the relationship between this change in metabolic enzyme localization and waves of transcriptional activity was confirmed. This study provided evidence that H_2_S increased PDC‐E1 S‐sulfhydration at Cys101 to regulate PDC‐E1 translocation from the mitochondria to the nucleus, thereby preventing VSMC proliferation under hyperglycaemic and hyperlipidaemic conditions. H_2_S may be a useful therapeutic strategy for cardiovascular diseases in the future.

## CONFLICT OF INTEREST

The authors declare that they have no conflict of interests.

## AUTHOR CONTRIBUTIONS

**Linxue Zhang:** Formal analysis (equal); Methodology (equal); Writing‐review & editing (equal). **Xiaoshu Jiang:** Formal analysis (equal); Methodology (equal); Writing‐review & editing (equal). **Ning Liu:** Investigation (equal); Validation (equal). **Mingyu Li:** Supervision (equal). **Jiaxin Kang:** Data curation (equal). **Lingxue Chen:** Data curation (equal). **Jingyuan Tang:** Visualization (equal). **Shiyun Dong:** Project administration (equal). **Fanghao Lu** **:** Conceptualization (equal); Funding acquisition (equal). **Weihua zhang:** Funding acquisition (equal); Writing‐original draft (equal).

## Supporting information

Fig S1

Fig S2

Fig S3

Fig S4

Fig S5

Supplementary Material

## Data Availability

All data generated during this study are included in this published article.
