# Supplementary figures and images for: Exogenous H2S prevents the nuclear translocation of PDC‐E1 and inhibits vascular smooth muscle cell proliferation in the diabetic state
Source: J Cell Mol Med. 2021 Aug 21;25(17):8201–14. doi: 10.1111/jcmm.16688 (PMC8419187; doi:10.1111/jcmm.16688)

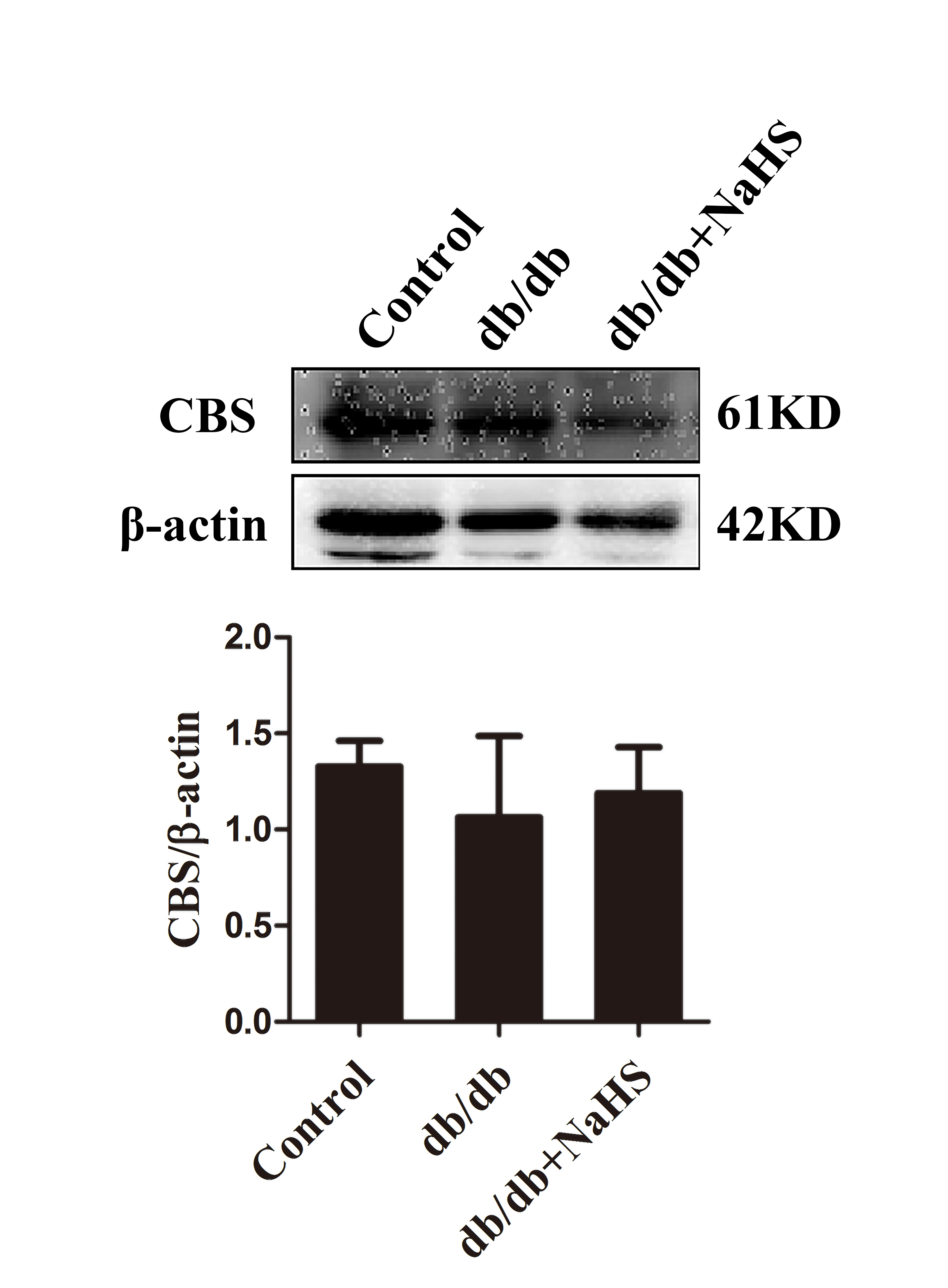

Supplement: Supplementary file 1 — Fig S1 [file JCMM-25-8201-s003.jpg]

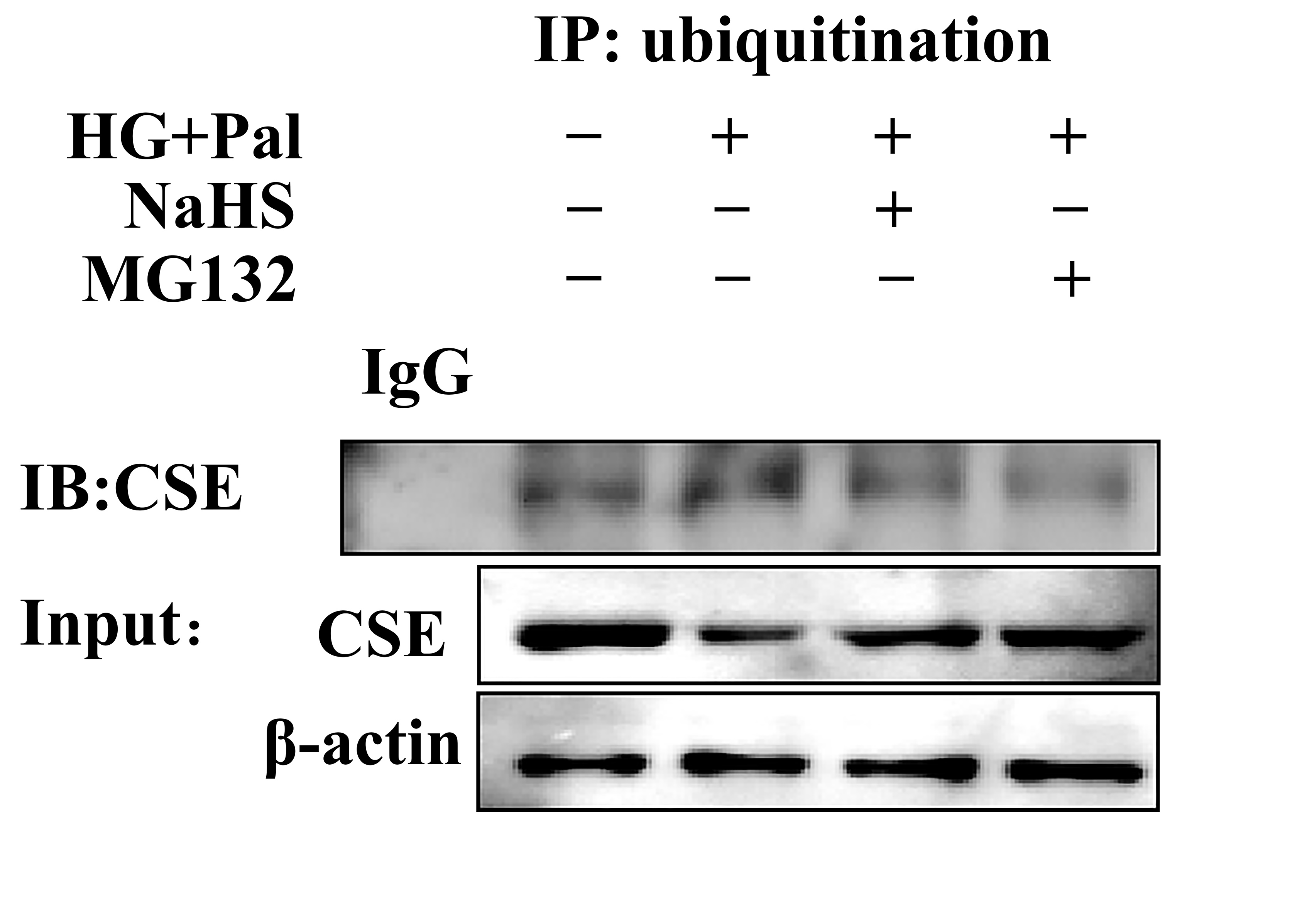

Supplement: Supplementary file 2 — Fig S2 [file JCMM-25-8201-s005.jpg]

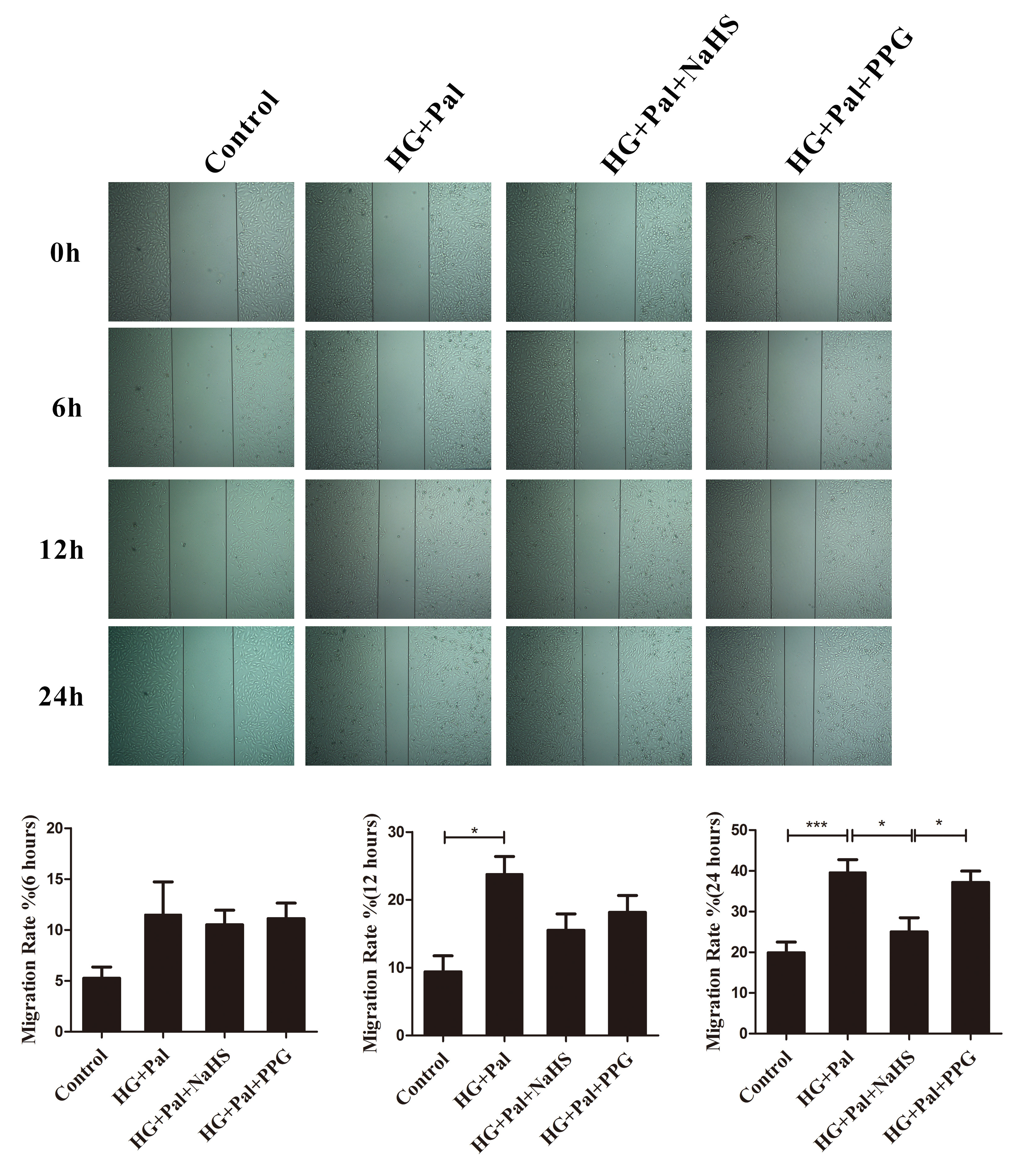

Supplement: Supplementary file 3 — Fig S3 [file JCMM-25-8201-s004.jpg]

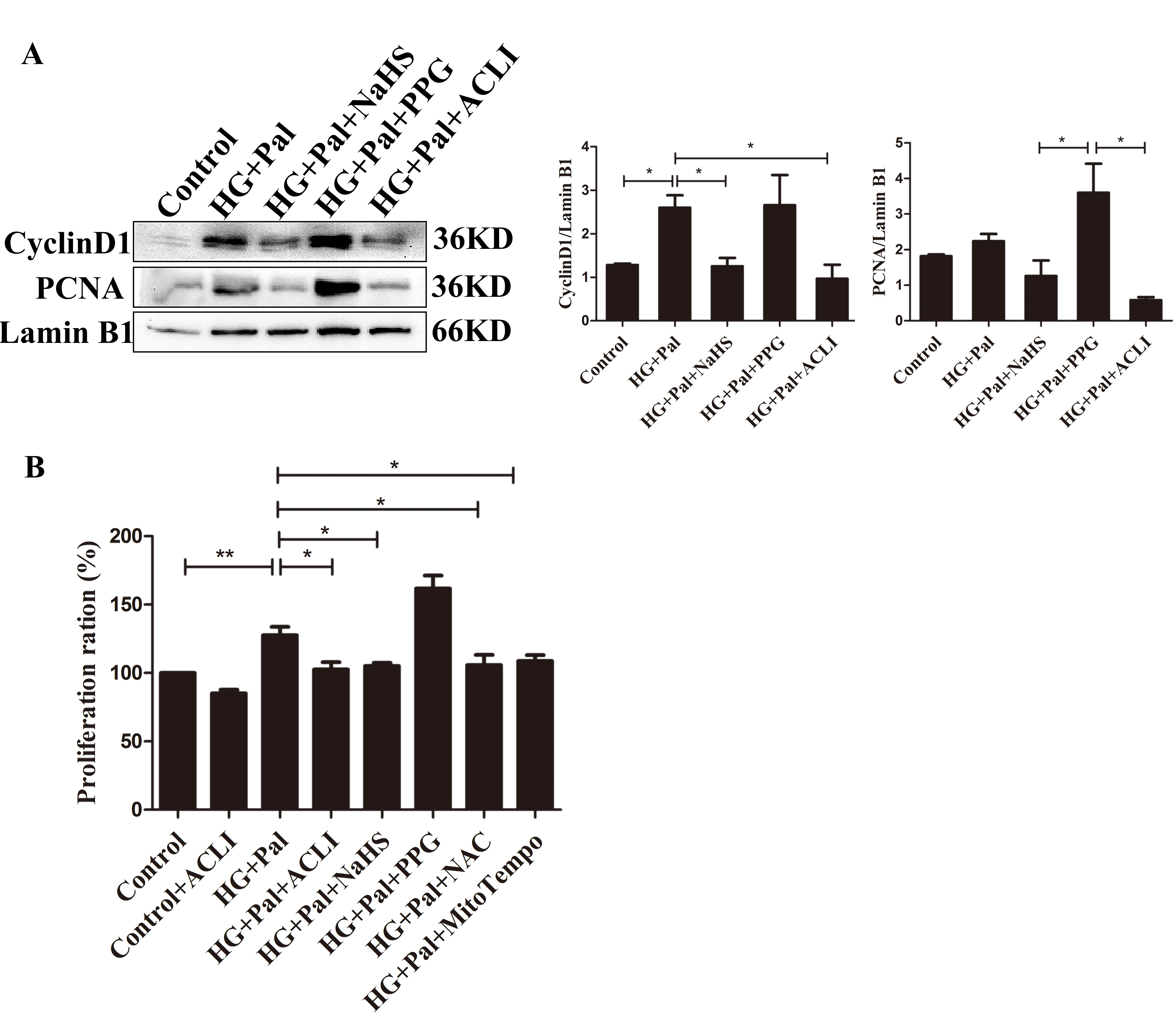

Supplement: Supplementary file 4 — Fig S4 [file JCMM-25-8201-s001.jpg]

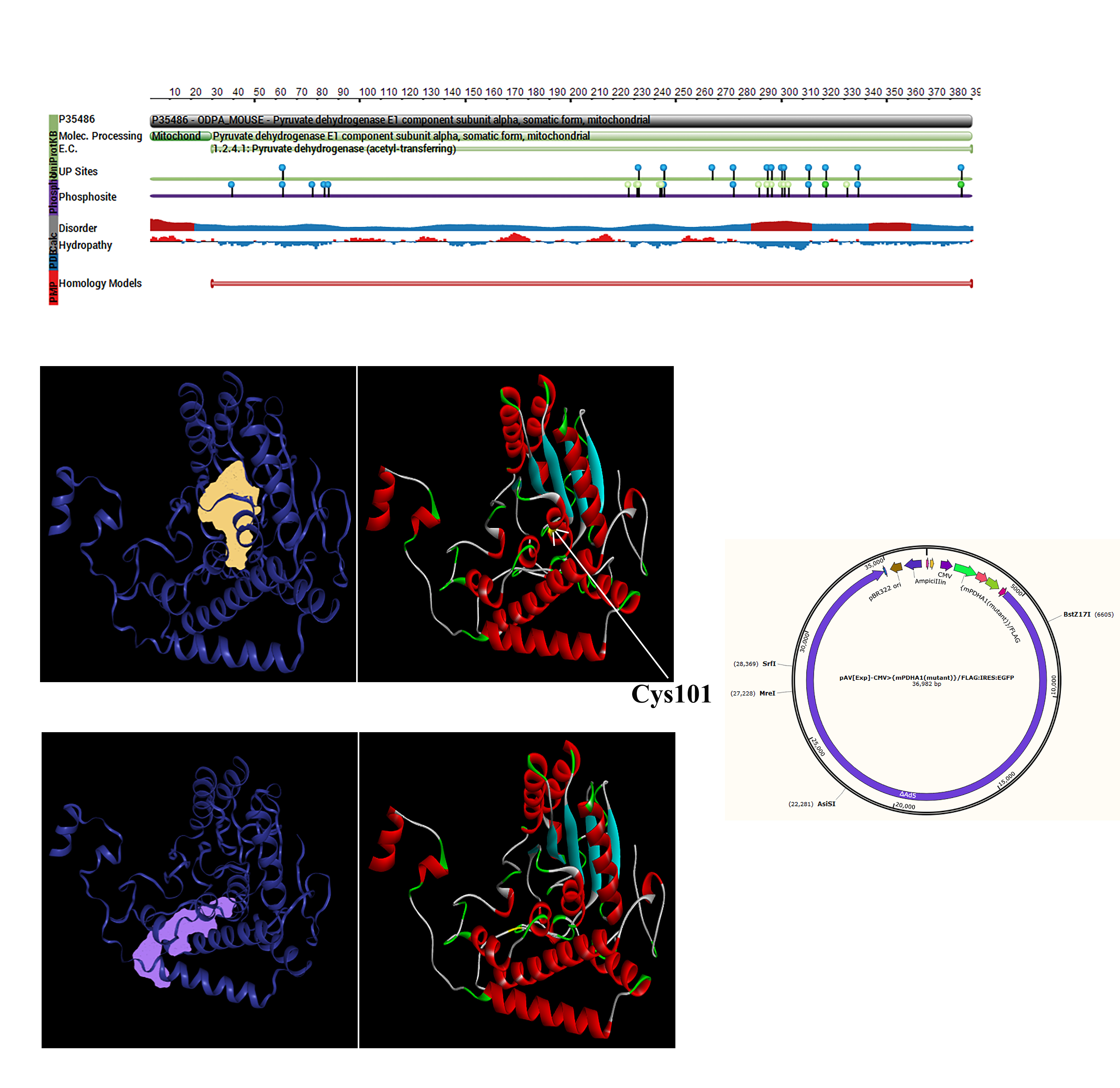

Supplement: Supplementary file 5 — Fig S5 [file JCMM-25-8201-s002.jpg]
