# Supplementary material for: Exogenous H2S prevents the nuclear translocation of PDC‐E1 and inhibits vascular smooth muscle cell proliferation in the diabetic state
Source: J Cell Mol Med. 2021 Aug 21;25(17):8201–14. doi: 10.1111/jcmm.16688 (PMC8419187; doi:10.1111/jcmm.16688)
Supplement: Supplementary file 6 — Supplementary Material [file JCMM-25-8201-s006.doc]

**SFigure 1.** Western blotting was used to detect the expression of CBS in the thoracic aortas of control mice, db/db mice and animals that received an intraperitoneal injection of NaHS (n = 4).

**SFigure 2.** Immunoprecipitation experiment to verify the interaction between ubiquitinated proteins and CSE.

**SFigure 3.** After stimulation with HG and palmitate for 6, 12 and 24 h, migration assays were performed (n = 4). Values are presented as the means ± S.D. **P* < 0.05 and ****P* < 0.001.

**SFigure 4.** The ATP citrate lyase inhibitor (ACLI, 50 μM) inhibited cell proliferation.

1. Western blotting was used to detect the expression of CyclinD1 and PCNA in the different experimental groups (n = 4).
2. CCK-8 assays were performed to determine the effects of different drug treatments on cell viability (n = 6).

Values are presented as the means ± S.D. **P* < 0.05.

**SFigure 5.** The active centres of PDC-E1 were predicted using a computational method.
